# Cooled radiofrequency ablation of the genicular nerves for chronic pain due to osteoarthritis of the knee: a cost-effectiveness analysis compared with intra-articular hyaluronan injections based on trial data

**DOI:** 10.1186/s12891-022-05445-z

**Published:** 2022-05-24

**Authors:** Mehul J. Desai, Anthony Bentley, William A. Keck

**Affiliations:** 1 International Spine, Pain & Performance Center, Washington DC, USA; 2 George Washington University, School of Medicine & Health Sciences, Washington DC, USA; 3 Mtech Access, Bicester, Oxfordshire UK; 4 Avanos Medical, Alpharetta, GA USA

**Keywords:** Cost-utility analysis, QALY, Economic analysis, CRFA, Hyaluronan, Osteoarthritis

## Abstract

**Background:**

Effective symptom control in painful knee osteoarthritis (OA) may improve patient quality of life. In a randomised crossover trial (NCT03381248), COOLIEF* cooled radiofrequency ablation (CRFA) reduced pain and stiffness and improved physical function and quality of life compared with intra-articular hyaluronan (HA) injections. The present study aimed to establish the cost effectiveness of CRFA versus intra-articular HA injections for treating moderate-to-severe OA knee pain from a US Medicare perspective.

**Methods:**

We conducted a cost-effectiveness analysis using utility data (EQ-5D) from the randomised crossover trial of CRFA versus intra-articular HA injections, which had follow-ups at 1, 3, 6, and 12 months. Patients in the HA group with unsatisfactory outcomes (e.g., continued pain) at 6 months could cross over to CRFA. Economic analysis outcomes included quality-adjusted life-years (QALYs), costs, and cost effectiveness (cost per QALY gained). Base-case analyses were modelled on a 6-month time horizon (to trial crossover). Due to limited trial data in the HA arm beyond 6 months, scenarios explored potential outcomes to 12 months if: 1) Utility with HA persisted for a further 6 months; 2) A second HA injection was received at 6 months and achieved the same utility change for the second 6 months. In both scenarios, the CRFA arm used trial data for patients who received CRFA from baseline to 12 months. Alternative costing scenarios were also explored.

**Results:**

CRFA resulted in an incremental QALY gain of 0.020 at an incremental cost of US$1707, equating to an incremental cost-effectiveness ratio (ICER) of US$84,392 per QALY over 6 months, versus intra-articular HA injections. Extending the analysis to 12 months and assuming persistence in utility in the HA arm resulted in a larger utility gain for CRFA (0.056 QALYs) and a lower ICER of US$30,275 per QALY. If patients received a second HA injection, the incremental benefit of CRFA out to 12 months was reduced (QALY gain 0.043) but was offset by the costs of the second HA injection (incremental cost US$832). This resulted in an ICER of US$19,316 per QALY.

**Conclusions:**

CRFA is a cost-effective treatment option for patients with OA-related knee pain considering the typical US threshold of US$100,000/QALY.

## Background

Knee osteoarthritis (OA) is characterised by pain, stiffness, and loss of function, which negatively impact on health-related quality of life [1, 2]. Symptomatic knee OA is estimated to affect approximately 14 million people in the US [3]. In addition to the patient burden, knee OA is a substantial economic burden, with estimated annual healthcare costs ranging from US$5.7 billion to US$15.7 billion [4], and annual absenteeism costs of US$10.3 billion [5].

Knee arthroplasty (including total knee arthroplasty [TKA]) is an effective and established terminal therapeutic option for late-stage OA-related pain and dysfunction [6]. However, arthroplasty may not be appropriate for all patients due to age, comorbidities, lack of social support, or other factors [7–10]. Furthermore, approximately 20% of patients remain dissatisfied following TKA, suggesting that it is not fully effective in all patients [11–13]. Continued pain post-TKA has also been reported in 8–34% of patients [14] and an estimated 3.8% require subsequent revision surgery [15]. Intra-articular injections of steroids (IAS) or hyaluronan (HA) may be used to manage symptoms. However, their efficacy appears to be limited; IAS offers only short-term pain relief [16] and may accelerate knee OA progression [17]. HA injections may offer modest reductions in pain over placebo [18] but these findings could not be verified in larger statistical analyses [19].

Outpatient delivery of radiofrequency ablation (RFA) to targeted genicular nerves represents a realistic, minimally invasive procedural option for patients with pain related to knee OA [20–22]. In particular, the COOLIEF* Cooled Radiofrequency (CRFA) System (Avanos Medical, Alpharetta, GA, USA), a cooled form of RFA with internally irrigated probes to optimise power transfer to target tissues, is an effective and safe long-term therapeutic option for improved pain management, physical function, and health-related quality of life in patients with knee OA [22–27]. To date, COOLIEF* is the only radiofrequency treatment to be approved by the US Food and Drug Administration for the management of OA knee pain [28].

CRFA has demonstrated clinical efficacy and cost effectiveness versus IAS in the management of symptomatic knee OA. In a randomised, controlled, open-label, multicentre, crossover trial (NCT02343003), CRFA significantly reduced knee pain versus IAS (*p* < 0.0001) at 6 months, with improvements sustained to 12 months [22, 23]. Significant improvements in the Oxford Knee Score (OKS) were also observed at 6 and 12 months post-treatment [22, 23]. These findings were applied in an economic analysis, resulting in incremental cost-effectiveness ratios (ICERs) of US$18,773 (6-month time horizon) and US$7462 (12-month time horizon) per quality-adjusted life-year (QALY) gained with CRFA versus IAS [29]. CRFA is therefore a highly cost-effective treatment option for symptomatic knee OA, considering the US$100,000/QALY threshold typically used in the US [30].

CRFA has also shown clinical efficacy versus intra-articular HA in the management of symptomatic knee OA. In a prospective, randomised, multicentre, crossover study (NCT03381248), CRFA reduced knee pain (measured by numeric rating scale [NRS]) by ≥50% in 65.2% of participants at 12 months [27]. Among patients who received HA, only 38% reported ≥50% pain relief at 6 months [31]. CRFA also conferred statistically significant improvements in the Western Ontario and McMaster Universities Osteoarthritis Index and EQ-5D-5L scores at 12 months. Furthermore, participants who were originally randomised to HA injection and who crossed over to CFRA at 6 months (*n* = 68) subsequently achieved statistically significant and clinically relevant improvements in pain, function, and health-related quality of life scores [27].

To the best of our knowledge, no studies have reported economic evaluations of CRFA versus HA for symptomatic knee OA. The current study aimed to evaluate the cost effectiveness of CRFA compared with intra-articular HA injection for moderate-to-severe pain due to knee OA from the US Medicare system perspective. A decision-analysis model was developed using outcomes from the clinical trial comparing CRFA with HA (NCT03381248) [27] and costs from routine practice.

## Methods

### Economic analysis overview

The present analysis was funded by Avanos Medical, of which one of the authors (WK) is an employee. The methodology for this analysis is similar to that reported previously in a comparison of CRFA versus IAS [29]. The cost-effectiveness analysis was developed in Microsoft Excel to evaluate the costs and health outcomes of patients undergoing CRFA or HA. The analysis was based on the clinical trial NCT03381248 [27] and mirrored the trial in terms of the interventions compared, the time horizon considered, the procedures performed, and the settings of care in which patients were managed. Comparative EQ-5D data from the trial were used to determine mean health gains achieved by patients undergoing each therapy in terms of QALYs. Costs were estimated from the US Medicare perspective. The primary outcome was the cost per QALY gained, which captures both the health gains and healthcare costs associated with treatment. We calculated the ICER as the difference in total cost between the CRFA and HA injection, divided by the difference in QALYs. ICER values were calculated where CRFA resulted in health benefits (increased QALYs) at an increased total cost.

### Economic analysis design

Clinical study NCT03381248 was a prospective, randomised, multicentre, crossover trial and has been reported in detail by Chen et al. [27]. The population considered in the economic analysis reflected the population enrolled in the trial, which was patients with radiologically confirmed knee OA of grade 2–4 within 6 months prior to study screening and knee pain for ≥6 months that interfered with functional activities and persisted despite ≥3 months of conservative treatments. Other inclusion criteria were a positive response to a single GNB of the index knee (decrease in numeric pain scores of ≥50%) and a pain score of ≥6 on the 11-point NRS scale for the index knee.

A total of 260 subjects gave their consent to participate in the trial and 177 participants were randomised 1:1 to receive either CRFA (COOLIEF*, Avanos Medical, Alpharetta, GA, USA) (*n* = 89) of genicular nerves or a single intra-articular HA injection (Synvisc-One® [Hylan G-F 20], Sanofi, Bridgewater, NJ, USA) (*n* = 88). The ablation technique has been described in detail previously [22, 23]. Briefly, patients were placed in the supine position with the treatment knee slightly flexed. After anaesthetising the CRFA sites, a CRF introducer was placed at the appropriate locations. Accurate probe positioning at 50% depth of the femur and tibia was confirmed using true lateral fluoroscopic visualisation. A 4-mm, 18-gauge, internally cooled active tip electrode was then placed into the introducer needle and positioning again confirmed in the anteroposterior and lateral fluoroscopic views. Motor stimulation at 2.0 V was applied to establish that there were no muscular contractions and sensory stimulation at < 0.5 V was applied in all target locations (four sites in the trial by Chen et al. [27] but commonly three or more) to reproduce concordant knee pain and ensure proximity of the probe to each of the target nerves (superomedial and inferomedial branches of the saphenous nerve and the superolateral branch of the femoral nerve). Each neural element was then anaesthetised with 1% lidocaine followed by CRFA at 60 °C for 150 seconds. Needles were then removed and patients were allowed to properly recover before being discharged home [22, 23].

**Fig. 1:**
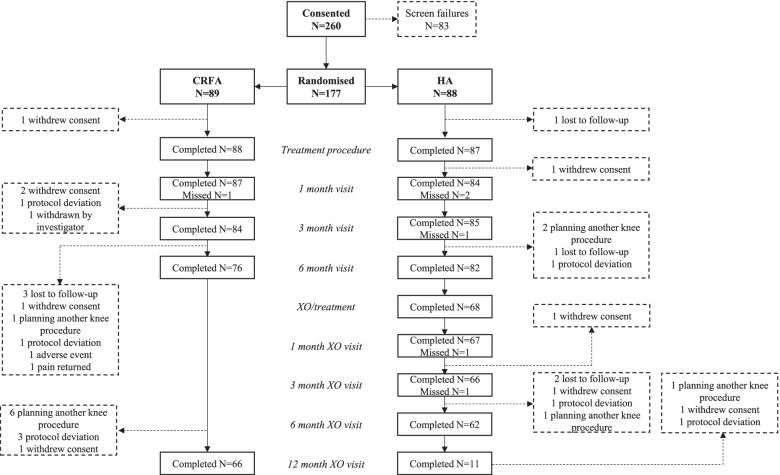
Patient flow in randomised controlled trial (adapted from Chen et al. 2020 [27])

Participants were assessed at study baseline and at 1, 3, 6, and 12 months post-intervention. The crossover design allowed participants who were deemed medically appropriate to choose to cross over and receive CRFA after the 6-month visit. Crossover participants were assessed at 7, 9, and 12 months post-baseline, corresponding to 1, 3, and 6 months post-CRFA treatment. In total, 76 participants in the CRFA cohort completed the 6-month follow-up and 66 participants completed the 12-month follow-up. Of the 88 patients in the HA cohort, 82 completed the 6-month follow-up. Of these participants, 68 (82.9%) chose to cross over and received CRFA and 62 completed the 6-month crossover follow-up. A total of 14 participants in the original HA cohort did not cross over to CRFA and 11 completed the 12-month follow-up [27]. Details of patient flow are presented in Fig. 1.

Our base case compared CRFA with HA using a time horizon of 6 months post-treatment, consistent with the interventions and follow-up periods within the trial. Scenario analysis considered a time horizon up to 12 months. The analysis did not extend beyond this timeframe, as to do so would have required assumptions to be made regarding the durability of the treatment effect and the need for retreatment, introducing uncertainty into the analysis. In line with the trial, our base case assumed that patients received one intervention with either CRFA or HA at study baseline, and that the benefits and costs included in the analysis reflected those associated with this baseline intervention. No repeat treatments were included in the base case analysis. Costs and benefits were not discounted for any analyses because the time horizon was only 6–12 months.

All patients screened for the trial underwent a single diagnostic genicular nerve block (GNB) to determine their eligibility for trial inclusion. A diagnostic GNB is part of the treatment algorithm for CRFA and involves fluoroscopy-guided injections of small volumes (0.60–0.75 mL at each site) of local anaesthetic. Pain scores were obtained before and after receiving GNB and patients experiencing a ≥ 50% decrease in NRS pain score were considered responders; these patients were eligible for study inclusion and randomised to their respective cohorts. Due to the high costs associated with GNB (Table 2), we included this screening test in our cost analysis. In clinical practice, this test would only be performed for patients who were to receive CRFA and not for those treated with HA; therefore, we applied the cost of GNB to the CRFA arm only.

### Clinical inputs and health utilities

The economic model calculated health benefits in the form of QALYs using trial-based changes in health-related quality of life, as measured using mean EQ-5D. EQ-5D is the most widely used utility measure employed in studies that estimate QALYs [32] and is preferred by health technology assessment bodies, such as the National Institute of Health and Care Excellence in the UK, and the Canadian Agency for Drugs and Technologies in Health [33, 34]. In the trial by Chen et al., EQ-5D values were collected at baseline, as well as the 1-, 3-, 6-, and 12-month/6-month crossover timepoints [27]. Absolute EQ-5D values were reported, which created an inherent bias in favour of CRFA due to a higher baseline utility for patients in the CRFA arm [27]. To adjust for this in the model, we assessed the relative change from baseline in EQ-5D values (Table 1).

**Table 1:** Utility scores (EQ-5D) used to model CRFA and HA in base case

	Baseline	Month 1	Month 3	Month 6
CRFA	0.66	0.79	0.80	0.79
HA	0.66	0.77	0.76	0.72

*Abbreviations*: *CRFA* Cooled radiofrequency ablation, *HA* Hyaluronic acid

### Costs

Costs were derived from Centers for Medicare and Medicaid Services fee schedules [35] and included standard physician (in-office or in-hospital) and hospital payments for HA, CRFA, and GNB procedures. The reference year for costs was 2020. All costs considered in the analysis were assumed to be accrued at the point when patients received their CRFA or HA intervention. In line with the trial protocol, we assumed that patients would not receive repeat treatment with CRFA, HA, or arthroplasty during the 6-month time horizon. Some participants in both treatment arms required opioid/non-opioid analgesics at baseline and during the treatment period. There were no statistically different changes from baseline in either opioid or non-opioid analgesia use during the study, although there was a trend towards reduced non-opioid medication use in the CRFA group at 12 months [27]. The costs of analgesia were therefore conservatively excluded from the analysis. We also assumed that patients would be discharged home with instructions for self-care following their treatment. In practice, patients in both treatment arms may have nurse follow-up visits and physiotherapy. As the care pathway is assumed to be the same for both treatment arms, costs of nurse and physiotherapy contacts were excluded from the analysis.

The base case reflected the care settings and procedures administered in the clinical trial [27]. For the CRFA procedure, the costs assumed that the procedure is performed in a non-office outpatient facility setting and involves ablation of the index knee at three anatomic locations using fluoroscopic visualisation of anatomic landmarks for accurate CRF probe placement. The number of anatomic locations is subject to between-study heterogeneity, with three nerves ablated in some studies and four in others (three is most commonly used although there is technically no maximum) [22, 23, 27, 31]. Importantly, the number of nerves that are ablated is not a cost modifier and reimbursement remains the same no matter how many locations are used. For the HA procedure, costs were estimated assuming that patients received one injection in the index knee under ultrasound guidance in a non-office outpatient facility setting. All trial participants underwent a single GNB to determine their potential to respond to CRFA and thus determine their eligibility for trial inclusion. This cost was therefore included in the analysis for all patients in the CRFA arm, assuming a non-office outpatient facility setting. Patients in the HA arm were assumed not to receive GNB, consistent with clinical practice. Summary costs are presented in Table 2.

**Table 2:** Total treatment costs applied in the economic analysis (US$)

	Base case	Scenario in-office GNB and HA
**CRFA procedure**	US$1872 (OP)	US$1872 (OP)
**HA procedure**	US$875 (OP)	US$648 (IO)
**GNB**	US$710 (OP)	US$218 (IO)

GNB costs are only applied in the CRFA arm, in line with clinical practice

*Abbreviations*: *CRFA* Cooled radiofrequency ablation, *GNB* Genicular nerve block, *HA* Hyaluronan, *IO* In-office, *OP* Outpatient

### Scenario analyses

Four scenario analyses were conducted to explore different assumptions regarding EQ-5D values between 6 and 12 months, and treatment costs.

Although EQ-5D data were collected at 12 months, we used a time horizon of 6 months in the base case. In the trial, only 14 patients remained on HA from 6 months onwards and only 11 of those patients returned to provide 12-month data, whereas 68 patients crossed over to receive CRFA [27]. Patients who crossed over were likely to have been non-responders to HA, whereas those who continued with HA were likely to have been responders to that treatment. The small number of patients who continued in the HA arm was deemed unsuitable to model the effect of HA between 6 and 12 months due to selection bias. Assumptions were therefore required in the HA arm after 6 months of treatment and these were explored in the scenario analyses with a 12-month time horizon:

- **Scenario 1:** The EQ-5D level in the HA arm persisted from 6 months onwards.
- **Scenario 2:** HA patients had a second injection and achieved the same absolute EQ-5D value as they did with the first injection.
- **Scenario 3:** A secondary cost analysis was conducted to account for any differences likely to be encountered in clinical practice. In this costing scenario, a 6-month time horizon was used, and it was assumed that GNB and HA were administered in an office setting while CRFA was administered in a non-office outpatient facility setting; this scenario is likely to be most reflective of a real-world setting for CRFA, HA, and GNB procedures in US clinical practice. Summary costs for this scenario are presented in Table 2.
- **Scenario 4:** A final scenario analysis was conducted to model patients who cross over to CRFA following a non-response to HA. This scenario had a 12-month time horizon and assumed that HA patients crossed over to CRFA at 6 months.

### Data analysis and sensitivity analysis

Conclusions of this study are based on a US$100,000 per QALY threshold, which is the current benchmark published by the Institute for Clinical and Economic Review in the US [30]. An intervention is considered cost effective if the ICER falls below this threshold.

We quantified the uncertainty around the conclusions using a probabilistic sensitivity analysis, in which all parameters were varied independently. Costs were assumed to follow gamma distributions, assuming that the ±10% plausible range equalled the 95% confidence interval, and utility inputs were varied using a beta distribution defined by their mean and standard error. The results of the probabilistic sensitivity analysis were depicted on scatter plots on the cost-effectiveness plane, showing the distribution of ICERs generated from 10,000 replicates. In addition, cost-effectiveness acceptability curves depict probabilistic sensitivity analyses results by showing the probability that CRFA would be cost effective versus HA over a range of monetary values that a decision-maker may be willing to pay per QALY.

Participants were well-matched across the CRFA and HA trial arms, although those in the CRFA arm had a significantly higher BMI [27]. In the trial, the subgroup analysis of CRFA responders was performed according to OA severity [27] but CRFA was found to be effective across varying grades of OA. Therefore, we did not consider any patient subgroups in our economic analyses.

## Results

### Base case

At a time horizon of 6 months post-treatment, CRFA was associated with a 0.020 QALY gain and an incremental cost of US$1707 compared with HA, which resulted in an ICER of US$84,392 per QALY gained (Table 3).

**Table 3:** Base case, CRFA versus HA (6-month time horizon)

Intervention	QALYs	Incremental QALY gain	Costs	Incremental cost	ICER
HA	0.372	–	$875	–	–
CRFA	0.392	0.020	Single GNB: $710 CRFA: $1872	$1707	$84,392

*Abbreviations*: *CRFA* Cooled radiofrequency ablation, *GNB* Genicular nerve block, *HA* Hyaluronan, *ICER* Incremental cost-effectiveness ratio, *QALY* Quality-adjusted life-year

### Scenario analysis results

#### Scenario 1

Scenario 1 used a 12-month time horizon and assumed that EQ-5D levels in the HA group persisted from 6 months. In this scenario, the incremental QALY gain with CRFA was 0.056 with an incremental cost of US$1707 vs HA. This resulted in an ICER of US$30,275 for CRFA (Table 4).

**Table 4:** Scenario analyses, CRFA versus HA

Analysis	Incremental QALY gain	Incremental cost	ICER
Scenario 1: 12-month time horizon, EQ-5D levels in HA group persist from 6 months	0.056	US$1707	US$30,275
Scenario 2: 12-month time horizon, HA patients receive a second injection and get the same change in EQ-5D for the second 6 months	0.043	US$832	US$19,316
Scenario 3: 6-month time horizon, single office-based GNB and HA injection (assumes CRFA is administered in non-office outpatient facility setting)	0.020	US$1422	US$71,314
Scenario 4: 12-month time horizon, HA patients cross over to CRFA at 6 months	0.025	-US$875	CRFA is dominant

*Abbreviations*: *CRFA* Cooled radiofrequency ablation, *GNB* Genicular nerve block, *HA* Hyaluronan, *ICER* Incremental cost-effectiveness ratio, *QALY* Quality-adjusted life-year

#### Scenario 2

Scenario 2 used a 12-month time horizon and assumed that HA patients received a second injection and had the same change in EQ-5D for the second 6 months. In this scenario, the incremental benefit of CRFA out to 12 months was reduced (QALY gain 0.043) compared with Scenario 1 but was offset by the additional costs of the second HA injection (incremental cost US$832). This resulted in an ICER of US$19,316 for CRFA (Table 4).

#### Scenario 3

Scenario 3 is the most likely clinical scenario and used a 6-month time horizon, assuming that the GNB and HA were administered in an office setting while CRFA was administered in a non-office outpatient facility setting. In this scenario, the incremental QALY gain with CRFA was 0.020 with an incremental cost of US$1422. This resulted in an ICER of US$71,314 for CRFA (Table 4).

#### Scenario 4

Scenario 4 used a 12-month time horizon and assumed that HA patients crossed over to CRFA at 6 months. In this scenario, the incremental QALY gain with CRFA alone was 0.025 with a cost saving of US$875. This resulted in CFRA alone dominating HA followed by CRFA (Table 4).

### Sensitivity analysis results

**Fig. 2:**
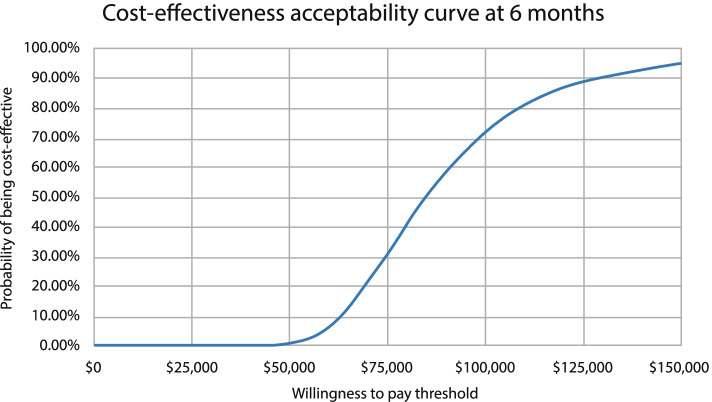
Cost-effectiveness acceptability curve at 6 months

The results of the probabilistic sensitivity analysis for CRFA versus HA at 6 months are presented in Fig. 2. At a US$100,000 per QALY threshold, CRFA has a 72% probability of being cost effective at 6 months (Fig. 2).

## Discussion

To the best of our knowledge, our study is the first to assess the cost effectiveness of CRFA versus HA for the treatment of knee pain due to OA. Using CRFA resulted in significant improvements in quality of life, as measured by EQ-5D, which translated into an ICER of US$84,392 per QALY at 6 months for CRFA versus HA. The ICER is below the threshold of US$100,000 per QALY recommended in the US [30], representing the maximum amount that a decision-maker may be willing to pay for the health benefits provided by the treatment. Sensitivity analysis demonstrated that the economic evaluation was robust to variation in data inputs, with a 72% chance that CRFA is cost effective versus HA at the US$100,000 per QALY threshold.

The effectiveness and safety of CRFA versus HA, as well as IAS in symptomatic knee OA has been well established in randomised clinical trials [22, 23, 27], while we have previously shown that CRFA is highly cost effective versus IAS over a 6-month time horizon with an ICER of US$18,773 per QALY [29]. This finding, together with the results of the current study, shows that CRFA is a cost-effective treatment option compared with these two alternative therapies. To validate this finding, future analyses should directly or indirectly compare CRFA with both IAS and HA (e.g., using a network meta-analysis). This approach was not possible for the current analysis due to the lack of directly comparable data. EQ-5D data were collected directly in the HA trial [27] and could be used to inform QALY calculations in the current study; however, this was not the case for the IAS study [22] and in that instance, OKS data had to be mapped to EQ-5D [29].

A 6-month time horizon was applied in the base case because only 14 patients in the clinical trial remained on HA from 6 months and of these patients only 11 returned to provide 12-month data [27]. Extending the time horizon to 12 months required assumptions regarding EQ-5D values in the HA arm, which were explored in multiple scenario analyses. Both 12-month scenarios showed considerable improvements in the ICER compared with the base case, which reflects the sustained benefit of CRFA versus HA. Furthermore, the assumption that the EQ-5D value in the HA arm persists from 6 to 12 months is potentially conservative given the apparent downward trajectory in EQ-5D with HA [27]. If patients received a second HA injection, the incremental QALY gained with CRFA was smaller than that in the first scenario, but was offset by the cost of the second injection.

All analyses included the cost of GNB, a diagnostic tool used to determine the potential responsiveness to ablation. Although GNB was used for all trial participants irrespective of therapy received (CRFA or HA) [27], it would not have been appropriate to include the costs of GNB in the HA arm in our analysis. As such, we assumed that only patients in the CRFA arm received GNB and accrued the costs. While discussions continue about the need for one or two GNBs, recent data reaffirm that favourable, consistent, and durable treatment outcomes can be achieved following a single GNB with ≥50% relief [22, 27, 36]. Modification of the cost assumptions to reflect real-world clinical practice, including in-office HA administration without ultrasound guidance (versus an outpatient facility using ultrasound) and in-office GNB for CRFA patients (versus outpatient consultation), did not change the overall conclusion that CRFA is cost effective versus HA.

The final scenario analysis included patients who received HA and then crossed over to CRFA at 6 months. In this scenario, CRFA was the dominant treatment strategy. These findings show that it is more cost effective to give CRFA in the first instance, rather than giving HA first and switching to CRFA in the event of a non-response.

In our analysis, we used trial data from a US multicentre study and took a US Medicare cost perspective. We are not aware of any evidence to suggest that the health benefits experienced by patients undergoing CRFA relative to HA may vary across geographic populations. However, differences in healthcare settings, clinical practice, and associated costs mean that further research is required to assess cost-effectiveness in individual markets.

There were several limitations to the current study that should be considered when interpreting the results. First, longer-term comparative clinical studies would allow cost-effectiveness estimates beyond 6–12 months to be generated. Second, missing data were not accounted for in the analysis. If the missing data are accounted for using a last observation carried forward analysis (LOCF), the ICERs increase but remain within the US$100,000 per QALY threshold for two scenarios: 1) 12-month time horizon, assuming constant EQ-5D in the HA arm from 6 months; and 2) 12-month time horizon, assuming second HA injection at 6 months. However, LOCF analysis of the base case (6-month time horizon) results in an ICER of US$164,880 per QALY. Third, we excluded costs other than screening and treatment, which is consistent with the trial where all participants were discharged home with self-care instructions. Follow-up costs, such as nurse follow-up and physiotherapy, may be expected, but the improved effectiveness and durability of CRFA compared with HA mean that these costs would likely be higher for HA-treated patients. No significant differences in analgesia use between the trial arms were observed, although there was a trend towards a reduction in non-opioid pain medication use in the CRFA group at 12 months [27]. Overall, these cost exclusions mean that the current analysis is likely to be conservative.

## Conclusions

In patients with symptomatic knee OA, CRFA offers health-related quality of life gains compared with conservative therapy with HA injections. From the US Medicare perspective, CRFA is an efficacious and cost-effective treatment option for patients with OA-related knee pain.

## Data Availability

In addition to the data inputs included in this published article and its supplementary information files, all data generated or analysed during this study are available from the corresponding author on reasonable request. This study and its results have not been presented elsewhere.

## Abbreviations

CRFA: Cooled radiofrequency ablation
GNB: Genicular nerve block
HA: Hyaluronan
IAS: Intra-articular steroid
ICER: Incremental cost-effectiveness ratio
LOCF: Last observation carried forward
OA: Osteoarthritis
QALY: Quality-adjusted life-years
RFA: Radiofrequency ablation
TKA: Total knee arthroplasty
UK: United Kingdom
US: United States
